# The relationship between height and fruit/vegetable intakes in adults: A nationwide cross-sectional study

**DOI:** 10.1177/02601060221108152

**Published:** 2022-06-14

**Authors:** Rafaela Rosário, Cesar Agostinis-Sobrinho, Patrícia Padrão, Oscar Lopes, Pedro Moreira

**Affiliations:** 1 School of Nursing, 56059 University of Minho, Portugal; 2 Health Sciences Research Unit: Nursing (UICISA: E), Nursing School of Coimbra (ESEnfC), Portugal; 3 Research Center in Child Studies, 56059 University of Minho, Portugal; 4 Research Centre in Physical Activity, Health and Leisure, Faculty of Sport, University of Porto, Portugal; 5 87241 Faculty of health Sciences - Klaipeda University – Lithuania; 6 Physical Education, Physiotherapy and Dance, Federal University of the South of Brazil, Porto Alegre, Rio Grande do Sul, Brazil; 7 Faculty of Nutrition and Food Sciences, University of Porto, Portugal; 8 Institute of Public Health, University of Porto, Portugal; 9 EPlural, CRL, Braga, Portugal

**Keywords:** Fruit, vegetable, adults, body height, cross-sectional

## Abstract

**Background:** Worldwide, fruit and vegetable intake is below recommendations. There is increasing evidence to suggest an association between height and diet. **Aim:** to analyse the associations of fruit and vegetable intake with attained height in Portuguese adults. **Methods:** A representative sample of 17 480 Portuguese adults (56.7% women) participated in this cross-sectional study. The survey comprised sociodemographic characteristics and self-reported height and weight. We performed regression models to assess the associations between height and fruit and vegetable consumption. **Results:** Compared with no intakes, those men with higher fruit and vegetables intake had 0.54 cm (95% CI 0.04; 1.04) greater height. Also, women with higher intakes of vegetables, fruit and vegetables combined were directly associated with a greater height of 0.29 cm (95% CI 0.03; 0.56 in vegetables) and 0.51 cm (95% CI 0.09; 0.93 in fruit and vegetables combined). **Conclusion:** Greater consumption of fruit and vegetables was directly associated with higher height in adults. From a health promotion perspective, intervention programmes aiming at improving fruit and vegetable intake should be highlighted so that full height potential is achieved.

## Introduction

Height and body mass index (BMI) are proxy anthropometric measures of the quality of cumulative net nutrition and healthiness of the living environment (de Onis and Habicht, 1996). They are predictive of health and developmental outcomes throughout life (Adair et al., 2013). Evidence suggests that taller people are considered in an advantage compared with shorter (NCD Risk Factor Collaboration (NCD-RisC), 2016; Tyrrell et al., 2016). Average adult height has steadily increased in a short period in high-income countries (NCD Risk Factor Collaboration (NCD-RisC), 2016; NCD Risk Factor Collaboration (NCD-RisC), 2020). Improvements in access to food, dietary diversity, sanitation, water, living standards, and lower exposure to disease may explain these trends (Fogel and Grotte, 2011; NCD Risk Factor Collaboration (NCD-RisC), 2020). Under this perspective, adult height may indicate overall health over time, environment, and cumulative healthy dietary intake (German et al., 2020).

Fruit and vegetables are an essential part of a healthy diet, particularly for their recognised capacity to modulate nutritional status and diseases (Silventoinen, 2003) the two most critical non-genetic determinants of adult height. These foods have a large content of micronutrients, dietary fibre and phytochemicals (FAO, 2015). Also, they exhibit pleiotropic and synergistic effects in combination, mainly antioxidant (Fiedor and Burda, 2014) and anti-inflammatory activities (Almeida-de-Souza et al., 2017; Vanamala, 2017). Yet, despite the recognised importance of fruit and vegetable consumption, in Portugal, as in other industrialised countries, the frequency of inadequate consumption is very high in infancy (Pereira-da-Silva et al., 2016), adolescence (Rosario et al., 2018b) and adulthood (Franchini et al., 2013).

Earlier evidence suggests that fruit and vegetable intake is associated with beneficial effects on the metabolic profile of people with overweight and obesity (Kopf et al., 2018; Williams et al., 2017). Furthermore, the consumption of these foods is associated with a lower likelihood of non-communicable diseases, including metabolic syndrome (Beydoun et al., 2019), overweight and obesity (te Velde et al., 2007), cardiovascular diseases (Miller et al., 2017), some types of cancer (Boeing et al., 2012), and all-cause of mortality (Wang et al., 2014). A recent systematic review found a positive influence of a dietary pattern characterised by fruit and vegetables on mental health, mainly in women (Guzek et al., 2021).

Despite the published evidence about changes in adult height across time (Padez, 2003; NCD Risk Factor Collaboration (NCD-RisC), 2020; NCD Risk Factor Collaboration (NCD-RisC), 2016), there is a lack of integration of dietary intake, mainly fruit and vegetable consumption, on adult height. We know that genetic factors influence dietary consumption, including coffee (Zhong et al., 2019; Cornelis et al., 2015), fish (Mozaffarian et al., 2017) and vegetables (Matoba et al., 2020). These studies highlight the influence of genetic heritability on dietary habits to a far greater magnitude than previously estimated. The brain is influenced by various signals that affect eating behaviours over time and underscores the genetic susceptibility for preferring some foods (Merino et al., 2022). With this study, we aim to analyse the associations of fruit and vegetable intake with attained height in a representative sample of Portuguese adults. We follow the hypothesis that there are direct associations between fruit and vegetable intake and attained height.

## Methods

### Participants and study design

This study gathered data from the Fifth Portuguese National Health Survey (NHS), developed by the national statistics institute. Sampling procedures included selecting participants from households between September and December 2014, using a multi-stage random probability design. The sample followed a stratified and multi-stage sampling scheme by NUTS II regions or subregions (1989 and 2002). The primary sampling units, consisting of one or more adjacent cells of the 1 km^2^ Grid INSPIRE, were systematically selected with a proportional likelihood to the size of the number of main household housing units. Then, secondary units (accommodation) were chosen at random and systematically within the first stage units. Finally, a primary sampling unit of 1 183 and 22 538 household units was gathered and randomly selected within each territorial unit.

A representative sample of 18 204 participants (56.4% women) with 15 years old and more was gathered, distributed by main Portuguese territorial units, such as North, Centre, Lisbon, Alentejo, and Algarve, as well as Madeira and Azores archipelagos. Trained staff interviewers performed the assessment, which comprised a questionnaire about social and demographic characteristics, health and chronic diseases such as obesity. We included only adults in the current manuscript, with a final sample of 17 480 adults (56.7% women).

### Anthropometry, sociodemographics and dietary intake

Height and weight were self-reported by adults, and we computed BMI (weight (kg)/height^2^ (m)). Also, participants’ age, sex, education level, family income per month and dietary intake were gathered. Level of education included the following categories: none, pre-school (0–6 years old), elementary school (1–6 years of education), middle school (7–9 years of education), high school (10–12 years of education) and higher education (more than 12 years). The categories of family income per month were defined as quintiles of income.

The NHS dietary questionnaire included information about fruit and/or vegetable intake, as follows: “How many portions a day of fruit do you usually eat?” and “How many portions a day of vegetable do you usually eat?”. These variables were recoded as “none”, “between 1 and 2 portions/day” and “3 and more portions/day”. Also, fruit and vegetables (F&V) intake was computed as the sum of portions of fruit and vegetables and further recoded as “none”, “between 1 and 4 portions/day”, and “five and more portions/day”.

Family structure was asked and recorded as follows: i) couple living with children under 25 years old; ii) couple living with no children under 25 years old; iii) single parent living with children under 25 years old, and iv) other types.

### Ethics

All procedures performed in the study were following the ethical standards of the national research committee and with the 1964 Helsinki declaration and its later amendments or comparable ethical standards. Data were anonymised and irreversibly de-identified to protect participants.

### Statistical analysis

Data are described as mean ± standard deviation (SD) for normally distributed variables or proportions where appropriate, conducted with sampling weights. Differences between subjects were analysed using a t-test for continuous variables and chi-squared for nominal and ordinal variables.

Associations of fruit, vegetable and F&V consumption with attained height were performed using generalised linear models (GLM). The models were further adjusted for age, level of education, BMI, family structure and family income. Since there were significant interactions between fruit and/or vegetables intake and height in men and women, the final analyses were performed separately by sex. Data analyses were performed using SPSS, version 26.0 (SPSS Inc. Chicago, IL), with a 0.05 level of significance considered.

## Results

Sociodemographic characteristics of the 17 480 participants are summarised in table 1. The national prevalence of obesity is 16.7%, significantly higher in women. Most participants were aged 40 to 64 years, 45.6% of whom had no formal or only elementary education. In addition, 31.4% of the families were composed of parents and children under 25 years old. Most participants ate between 1 and 2 portions of fruit and vegetables (47.3% and 47.8%, respectively) and between 1 and 4 portions of F&V (61.6%).

**Table 1.** Selected characteristics of the national health survey- Portugal.

			Weighted %		
	N	^N	Weighted %	Women	Men
Participants	17 480	8 332 208	100	53.5	46.5
Age					
20–39	4 054	2 575 015	30.9	29.4	32.6
40–64	7 725	3 652 026	43.8	43.0	44.8
65–84	5 090	1 843 570	22.1	23.6	20.4
≥85	611	261 597	3.1	4.0	2.1
Education					
None	2 349	812 174	9.7	13.3	5.7
Elementary school	7 235	2 991 917	35.9	33.8	38.3
Middle school	3 077	1 381 207	16.6	13.9	19.6
High school	2 895	1 631 243	19.6	18.3	21.0
Higher education	2 648	1 515 667	18.2	20.7	15.3
Family income					
1^st^ quintil	3 788	1 611 899	19.3	21.2	17.2
2^nd^ quintil	3 590	1 640 707	19.7	20.7	18.6
3^rd^ quintil	3 458	1 670 153	20.0	20.9	19.1
4^th^ quintil	3 330	1 692 025	20.3	18.5	22.4
5^th^ quintil	3 314	1 717 424	20.6	18.7	22.8
Family structure					
Couple with children under 25 years old	4 257	2 616 986	31.4	30.2	32.8
Couple without children under 25 years old	4 681	1 995 831	24.0	22.2	25.9
Single parent with children under 25 years old	733	308 265	3.7	5.3	1.8
Other	7 809	3 411 125	40.9	42.2	39.5
BMI status					
Underweight and normal weight	7 204	3 735 460	44.8	48.3	40.9
Overweight	6 768	3 083 314	37.0	31.9	42.9
Obesity classes I	2 499	1 107 389	13.3	13.6	12.9
Obesity class II and III	690	286 809	3.4	4.3	2.4
Portions of fruit/day					
None	4 840	2 334 041	28.0	24.5	32.1
Between 1–2	8 383	3 937 770	47.3	49.0	45.3
3 and more	4 257	2 060 397	24.7	26.6	22.6
Portions of vegetables/day					
None	8 322	3 639 386	43.7	38.3	49.9
Between 1–2	7 700	3 978 994	47.8	52.2	42.6
3 and more	1 458	713 828	8.6	9.5	7.5
Portions of F&V/day					
None	3 519	1 637 050	19.6	16.7	23.0
Between 1–4	10 920	5 129 317	61.6	61.9	61.2
5 and more	3 041	1 565 841	18.8	21.4	15.8
Height (m) | mean (SD)	1.6 (0.1)				
Weight (kg) | mean (SD)	71.5 (13.6)				
BMI (kg/m^2^) | mean SD	26.3 (4.4)				

n sample size, ^N estimated population size.

Values are proportions (%) unless for weight, height and BMI which are mean (SD). Sample weighted pond 1.

After adjusting for age, level of education, BMI, family structure and income, in men, those who consumed five or more portions of F&V were directly associated with a 0.54 cm greater attained height (95% CI 0.04; 1.04). On the other hand, women who consumed between 1 and 2 portions of vegetables were directly associated with 0.29 cm greater height (95% CI 0.03; 0.56). Also, in women, each consumption between 1 and 4 portions of F&V and five and more portions of F&V (when compared with the reference no consumption) were directly associated with 0.5 cm (95% CI 0.19; 0.87 and 0.09; 0.93, respectively) greater attained height (table 2).

**Table 2.** Adult height (cm) according to fruit and/or vegetable intake in a Portuguese nationwide sample.

	All		Women		Men	
	Unadjusted (Crude b coefficient (95% CI)	Adjusted^1^ (Crude b coefficient (95% CI)	Unadjusted (Crude b coefficient (95% CI)	Adjusted^1^ (Crude b coefficient (95% CI)	Unadjusted (Crude b coefficient (95% CI)	Adjusted^1^ (Crude b coefficient (95% CI)
Fruit (portions/day)						
None	ref	ref	ref	ref		ref
Between 1–2	**−1.21 (−1.54; −0.89)**	0.15 (−0.08; 0.38)	−0.09 (−0.42; 0.24)	0.29 (−0.03; 0.60)	**−0.67 (−1.05; −0.29)**	−0.02 (−0.37; 0.33)
3 and more	**−1.26 (−1.64; −0.88)**	0.22 (−0.06; 0.49)	0.16 (−0.21; 0.54)	0.26 (−0.10; 0.62)	**−0.52 (−0.98; −0.06)**	0.27 (−0.15; 0.69)
Vegetable (portions/day)						
None	ref	ref	ref	ref	ref	ref
Between 1–2	**−0.80 (−1.09; −0.51)**	**0.25 (0.05; 0.45)**	**0.64 (0.36; 0.92)**	**0.29 (0.03; 0.56)**	0.21 (−0.14; 0.56)	0.17 (−0.14; 0.49)
3 and more	**−0.82 (−1.34; 0.31)**	0.21 (−0.15; 0.58)	**1.05 (0.58; 1.53)**	0.25 (−0.20; 0.70)	**0.77 (0.09; 1.45)**	0.21 (−0.40; 0.83)
F&V (portions/day)						
None	ref	ref	ref	ref	Ref	ref
Between 1–4	**−1.20 (−1.55; −0.85)**	**0.28 (0.04; 0.54)**	0.16 (−0.20; 0.52)	**0.53 (0.19; 0.87)**	**−0.70 (−1.10; −0.30)**	0.043 (−0.32; 0.40)
5 and more	**−1.36 (−1.81; −0.91)**	**0.43 (0.11; 0.75)**	**0.69 (0.25; 1.13)**	**0.51 (0.09; 0.93)**	0.05 (−0.50; 0.60)	**0.54 (0.04; 1.04)**

All values are Crude b coefficient (95% CI, 95% confidence intervals).

^1^ Generalised linear models adjusted for age, level of education, BMI, family structure, family income.

Significant associations are in bold type.

## Discussion

In this representative study, higher intakes of vegetables (in women) and F&V (both in men and women) were directly associated with a greater height after adjustment for age, level of education, BMI, family structure, and income. This is in accordance with a former study that found direct associations between fruit and vegetables intake with height attainment during childhood (Rosário et al., 2021). We know that current fruit and vegetables intake might differ from previous intakes. However, evidence suggests that there are biological and neural mechanisms that contribute to the interindividual differences about dietary intake (Merino et al., 2022; Matoba et al., 2020; Zhong et al., 2019; Cornelis et al., 2015; Mozaffarian et al., 2017). Furthermore, it is also possible that those who currently consume higher amounts of fruit and vegetables may have had a better nutritional environment during childhood (Mikkilä et al., 2004; NCD Risk Factor Collaboration (NCD-RisC), 2020), adolescence (te Velde et al., 2007), and had an overall healthier eating pattern (Ramsay et al., 2017; Hoy et al., 2020), contributing to growth in height (Schwingshackl et al., 2015; Ledoux et al., 2011).

The roles of fruit and vegetable may explain the observed associations with height attainment. Their consumption may decrease acidic load and support bone metabolism, particularly bone mass (Prynne et al., 2006; Movassagh et al., 2017; McGartland et al., 2004). Indeed, there is a greater calcium excretion during acidosis due to the release of alkaline components from the bone tissues that buffer the acid load (Barzel and Massey, 1998). However, exogenous buffers, such as fruit and vegetables (Barzel and Massey, 1998) and some nutrients highly prevalent in these foods (e.g. potassium and magnesium) (Welch et al., 2008), may contribute to a retainment of calcium. Furthermore, vitamins from plant-sourced foods, such as vitamin C (Aghajanian et al., 2015) and vitamin K (Weber, 2001), also benefit bone health.

The trends of height in the world are heterogeneous and vary according to each country's singularity. A previous Portuguese study found inverse associations between BMI categories and attained height in adults (Rosario et al., 2018a), although there are inconsistent results between height and obesity in the last decades (NCD Risk Factor Collaboration (NCD-RisC), 2016; NCD Risk Factor Collaboration (NCD-RisC), 2020). We know that fruit and vegetables are low in energy density, fat and have high water and dietary fibre content. They are both associated with higher satiety and consequently contribute to tackling overweight and obesity (Rolls et al., 2004). In the current study, the observed associations between fruit and vegetables intake and height are maintained even after adjusting for BMI, suggesting the latter is not a mediator in the currently found relationship.

This study has several strengths that should be acknowledged. First, we used a representative sample of Portuguese adults, allowing the generalisation to Portugal's adult population. Second, we performed the analysis accounting for important confounders, including family structure and income per month. For example, previous studies found direct associations between fruit and vegetable consumption and income and family structure (Oliveira et al., 2014). Third, we performed the analysis according to sex, accounting that women and men grow differently, with men achieving a higher height than women (NCD Risk Factor Collaboration (NCD-RisC), 2016).

The study also has limitations. First, weight and height were self-reported, and it is not possible to exclude recall bias. Previous studies found that older people may overestimate self-reported height (Kuczmarski et al., 2001), in contrast to the self-reported weight, which may be underestimated by men and women (Connor Gorber et al., 2007). Second, fruit and vegetable intake was collected based on people's usual intake questions. This approach could be considered less accurate than questionnaires considering the frequency and variety. We are aware that there is no perfect measurement of portions and dietary intake (Wrieden and Momen, 2009). Nevertheless, photographs, which were used in the current study, are considered portion-size measurement aids that facilitate the recognition of the portion that the person actually ate when comparing with the photographs (European Food Safety Authority, 2009; Wrieden and Momen, 2009). Still, there might be a trend to overestimate fruit and vegetable intake due to social desirability and social agreement bias (Miller et al., 2008). Finally, the study is cross-sectional, and we cannot establish causal relationships.

Further research is needed with longitudinal data to confirm or rule out our findings. Besides, intervention programmes aiming to improve fruit and vegetable consumption and height may be implemented with children and adolescents, who are still growing in stature, to achieve their full growth potential.

## Conclusion

Greater consumption of fruit and vegetables is directly associated with a higher height in adults. Therefore, community and public health should highlight intervention programmes to improve fruit and vegetable intake to achieve full height potential.
